# Comparative Approach of the *de novo* Fatty Acid Synthesis (Lipogenesis) between Ruminant and Non Ruminant Mammalian Species: From Biochemical Level to the Main Regulatory Lipogenic Genes

**DOI:** 10.2174/138920210791110960

**Published:** 2010-05

**Authors:** G.P. Laliotis, I. Bizelis, E. Rogdakis

**Affiliations:** Department of Animal Science, Laboratory of Animal Breeding and Husbandry, Agricultural University of Athens, Iera Odos 75,118 55 Athens, Greece

**Keywords:** Fatty acids, genes, lipogenesis, ruminants, sheep.

## Abstract

Over the second half of 20^th^ century much research on lipogenesis has been conducted, especially focused on increasing the production efficiency and improving the quality of animal derived products. However, many diferences are observed in the physiology of lipogenesis between species. Recently, many studies have also elucidated the involvement of numerous genes in this procedure, highlighting diferences not only at physiology but also at the molecular level. The main scope of this review is to point out the major differences between ruminant and non ruminant species, that are observed in key regulatory genes involved in lipogenesis. Human is used as a central reference and according to the findinggs, main differences are analysed. These findings could serve not only as basis for understanding the main physiology of lipogenesis and further basic research, but also as a basis for any animal scientist to develop new concepts and methods for use in improving animal production and modern genetic improvement.

## 1. INTRODUCTION

During the past decades consumers have started seeking animal products of low fat content and of high and consistent quality. This fact has led animal production to invest in research aimed at the study of metabolism and the procedure of lipogenesis.

Lipogenesis occurs in all vertebrate species and entails a number of metabolic steps that lead to the synthesis of fatty acids and subsequent triglyceride synthesis. The major sites that lipogenesis generally takes place are the intestinal mucosal cells, the liver, the adipose tissue and in lactating mammals the mammary gland. The intestinal mucosal cells have as a main role to handle and use the fatty acids absorbed from the diet. In contrast, the other three tissues are responsible for the *de novo* synthesis of fatty acids, using as a prior molecule the acetyl-coA derived from the catabolism of carbohydrates and to a lesser extent the amino acids.

In ruminants the predominant anatomic site for lipogenesis is adipose tissue [1]. Most of the dietary carbohydrate is fermented to acetate, propionate and butyrate in the rumen, so liver metabolism is dominated by glucose synthesis while acetate is the major lipogenic precursor in adipose tissue and in mammary gland during lactation [2]. On the contrary, in human and birds the liver is considered as the major site for lipogenesis, whereas pig seems to resemble the ruminants with respect to lipogenesis. In rodents and rabbit both liver and adipose tissue are important for synthesizing fatty [3,4].

**Fig. (1):**
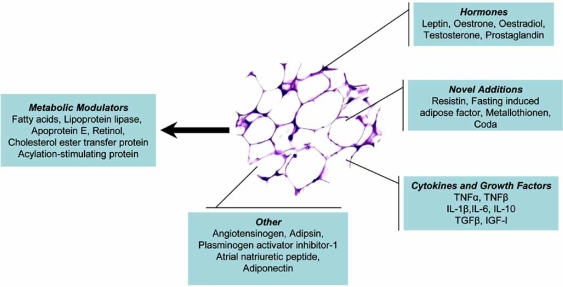
Regulator molecules secreted by adipose tissue.

The main key role of adipose tissue is the control of energy balance, providing the appropriate “fuel” in the form of fatty acids, when it is required. Adipose tissue can also offer a mechanical role and a thermal insulation [5]. This is particularly evident in the case of blubber observed in marine species [6]. Moreover, Cousin *et al.* [7] have reported the involvement of adipose tissue in inflammatory processes, as preadipocytes observed to act as macrophage-like cells. In addition to all these, adipose tissue plays an important role in glucose homeostasis. The discovery of leptin [8], a cytokine-like factor secreted from mature adipocytes, demonstrated that adipose tissue can works also as an endocrine and secretory organ [2,5,6]. Recently, many other substances apart from leptin were found to be secreted from adipose tissue (Fig. **1**), playing an important role in the regulation of energy balance and other physiological processes [see 2, 6, 9, 10]. Thus, the role of adipose tissue is far beyond the simple role of fat storage, ranging from the metabolic regulation to the physiological homeostasis.

In ruminants, apart from the above roles, adipose tissue growth, which results from a change in either fat cell size (hypertrophy) or fat cell number (hyperplasia) or both [11], can affect the economic return of the production system. Excess fat deposits influence negatively the grading of carcasses [11, 12]. Moreover, the high intramuscular fat deposition (marbling) is a desired characteristic of meat quality [13]. In addition, the dynamics of adipose tissue metabolism, especially during puberty or during the last stage of pregnancy, is related to health status (i.e. ketosis or toxaemia) and future reproductive and lactating performance [14]. Thus, the control of fatty acid synthesis (lipogenesis) is of utmost importance in a ruminant’s production system.

Several studies have been conducted in many eutherian species (human / animals) investigating aspects and factors influencing or controlling lipogenesis (diet, hormones, stress, age, breed etc.). The advent of new technologies at molecular level has elucidated many genes implicated in the complicated procedure of lipogenesis, providing new insights into understanding its function and offering the possibility to explore new perspectives in studies of lipogenesis. The main aim of this review is to elucidate any difference existing in the key regulatory genes involved in the *de novo* synthesis of fatty acids between ruminants (a group of mammals with a different metabolic pathway and main anatomic site for lipogenesis) and non-ruminant species, sketching out, at the same time, possible future expectations based on the above field. As a base line human species is set and according to this any discrepancies are further discussed.

## 2. LIPOGENESIS: A COMPLEX BIOCHEMICAL PROCEDURE IMPELLED BY MULTI-FACTORIAL STIMULI

### 2.1. Pathway

The metabolic pathways involved in the synthesis of fatty acids and their subsequent esterification to form triacylglycerols are well established and have been studied in considerable detail [15, 16]. Fatty acids are synthesized by an extramitochondrial system [17], which is responsible for the complete synthesis of palmitate from acetyl-CoA in the cytosol. A well recognized and major metabolic difference between the ruminant and non-ruminant is the failure of carbon from glucose to contribute to fatty acid synthesis within the tissues of a ruminant, including mammary gland. This phenomenon is usually account for by the low activity of ATP citrate lyase and malate dehydrogenase. In ruminants the most of glucose is derived from gluconeogenesis [18], while acetate, and in a less portion propionate and butyrate, which are the main fuel molecules produced in the rumen, compromising the precursors for the initiation of lipogenesis in both adipose tissue and mammary gland.

Acetate is transformed into pyruvate and then, *via* oxidation within mitochondria, is further transformed into acetyl-CoA. Acetyl-CoA is the principal building block of fatty acids. Fig. (**2**) displays the major biosynthetic steps involved in the formation of fatty acids. The biosynthesis of palmitate is catalyzed by the complex of fatty acid synthase (FAS). The overall reaction catalyzed by FAS can be summarized by the equation: 

Acetyl-CoA + 7Malonyl-CoA +14NADPH +14H^+^ ➔ Palmitate + 14NADP + 8CoA + 7CO_2_ ^+^ + 6H_2_O

The malonyl-CoA that is required is derived from the acetyl-CoA under the catalytic action of acetyl-CoA carboxylase. It should be noted that in small ruminants propionyl-CoA can be used in place of acetyl-CoA as the primer molecule for fatty acids synthesis giving rise to the odd-numbered of fatty acids [13, 19].

From the above reaction it is obvious that the biosynthesis of fatty acids apart from carbon substrate requires also considerable amounts of reducing equivelants in the form of NADPH for the reduction of acetyl-CoA to fatty acids [20, 21]. The principal enzymes that are responsible for the NADPH production in ruminants are: i) glucose 6-phosphate dehydrogenase (G6PD), ii) 6-phosphogluconate dehydrogenase (6PGD), iii) cytosolic NADP malate dehydrogenase (malic enzyme, ME1) and iv) cytosolic NADP isocitrate dehydrogenase (IDH1) 

**Fig. (2):**
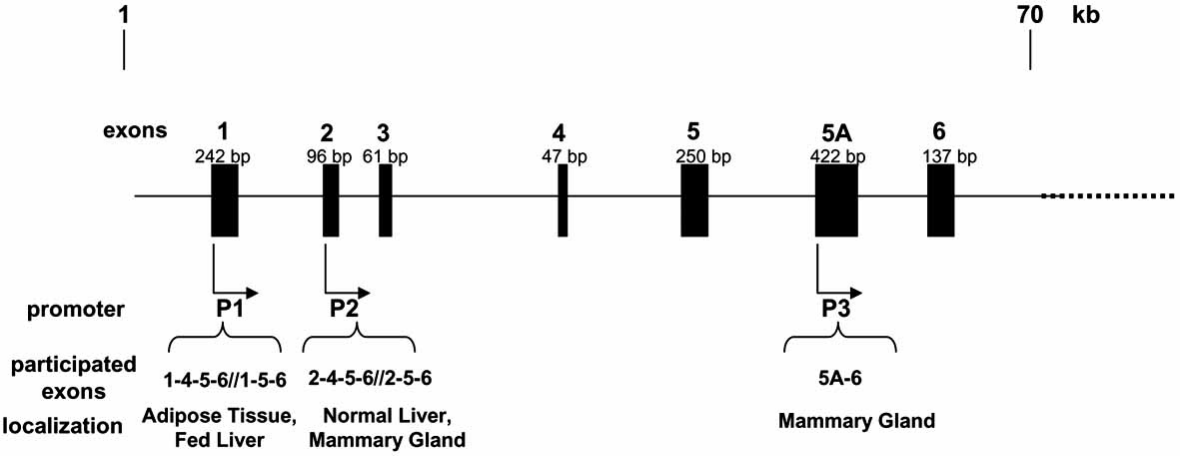
Ovine ACC transcripts formation [25]. Ovine ACC gene consists of three distinct promoter regions which give rise to different transcripts according to tissue.

The first two enzymes (G6PD and 6PGD) comprise the major sources of NADPH and they are involved in the pentose phosphate shunt. Specifically, G6PD catalyzes the first committed reaction, while 6PGD catalyzes the final reaction in the oxidative pentose pathway (Fig. **2**). Approximately the 30 to 50% of the required NADPH comes from the catalytic action of G6PD and 6PGD [12, 22]. In cow and sheep this percentage can reach the 50-100% and 30-100%, respectively [13]. NADP malate dehydrogenase is generally thought to have a minor role in NADPH production in ruminants rendering the predominant in non-ruminants pathway sequence of citrate➔ oxaloacetate➔ malate➔ pyruvate sequence not significant in ruminants [13]. Concerning IDH, in ruminants it has been suggested that it is responsible for providing the rest of NADPH not produced by the pentose phosphate shunt [1, 13]. Palmitate is then used as the substrate for further synthesis of fatty acids through the procedures of elongation and/or desaturation, which are taking place in the endoplasmic reticulum (ER) *via* the interaction of many catalytic enzymes (i.e. reductases, desaturases, elongases).

### 2.2. Factors Affecting Lipogenesis

Lipogenesis belongs to the group of quantitative traits. This means that apart from factors concerning the genetic make-up of the animal, many other non-genetic factors belonged to the so-called “wider micro/macro environment” can affect the procedure of lipogenesis.

A genotype is fixed at the moment of conception. Once it receives its genes, which are the direct link between parent and offspring, it is landed with those genes for life. However, it should be noted that different sets of genes are switched on and off under different environments. Breed has been used to provide a possible explanation of the observed difference in metabolic rates and in adipose tissue size in a variety of studies. The differences observed in fat distribution among several breeds of sheep and goats [for review see 23] are likely related to genetic differences in the metabolic regulators resulting in different lipogenic activity [12]. Meat type breeds have the ability to deposit more fat than the dairy breeds. According to Vernon [13] both subcutaneous and perirenal adipose tissue from beef cattle had greater rate of fatty acids synthesis than the same type of adipose tissue from dairy cattles of the same age and weight.

However, differences can also observed in breeds of the same productive type. For example, Panopoulou *et al.* [24] comparing two Greek dairy breeds, observed that the perirenal adipose tissue of the Karagouniki breed was more developed in respect with Chios breed at the same age and weight. Differences were also observed by the same authors, in the enzymatic activities of NADPH generating dehydrogenases in the adipose tissue of the above breeds, indicating the different fatness between the two breeds.

Analyzing the “wider environmental” factors, lipogenesis in all species is known to be subjected to acute homeostatic and to chronic homeorhetic control [25-27]. The former includes a variety of factors involving hormones (i.e. insulin) and locally produced modulators (i.e. prostaglandins). Homeorhetic control is concerned with modulation of lipid metabolism of tissues to meet the changing needs of a particular physiological, nutritional or pathologic state. The recently discovery that adipose tissue can produce many others molecules apart from fatty acids (i.e. leptin, TNFa, resistin) led to the formulation that lipogenesis is also susceptible to a third type of control called autonomic. This type of control refers to factors produced within the adipose tissue controlling lipogenesis and is concerned with modulating the mass of adipose tissue to meet the needs of animals [2]. However, analyzing further this topic is out of the purpose of this review.

## 3. FOCUSING ON MOLECULAR LEVEL

In order to be achieved either the homeostatic or homeorhetic and autonomic control, stimulation of the genes involved in the procedure of the *de novo* synthesis of fatty acids should be firstly take place. Thus, lipogenic gene’s expression is the first level for elucidating new mechanisms of control. Developments in molecular techniques have allowed elaborated studies of the genes, bringing to light novel levels of complexity concerning the regulation of lipogenesis. The scope of this section is the review of the advances in the main lipogenic genes isolated in ruminants and non ruminant mammalian species (especially human, mouse, rat), elucidating, in the possible extent due to data limitation, the complexities that have aroused. Specific attention is given to the major lipogenic genes encoding acetyl-CoA carboxylase, fatty acid synthase and NADP-dependent dehydrogenases.

### 3.1. Acetyl-CoA Carboxylase

Acetyl-CoA carboxylase (ACC), the rate-limiting enzyme for the biosynthesis of fatty acids, is a biotin containing cytoplasmic enzyme that catalyses the ATP-dependent carboxylation of acetyl-CoA to form malonyl-CoA, the activated donor of two carbon units for fatty acid chain elongation by the enzyme fatty acid synthase. Two isoenzymes of ACC, termed ACC-*α* and –*β*, transcribed from separate genes and with different functions have been described [27-30].

ACC-*α* gene gives rise to a 265 KDa enzyme that is ubiquitously expressed. Its expression is highly inducible in the major lipogenic tissues such as adipose tissue, liver and lactating mammary gland. Concerning ACC-*β* gene, it encodes a 275-280 KDa protein and is expressed predominately in tissues that utilize fatty acids as an energy source, i.e. heart and skeletal muscle [30-32]. Interestingly, it does not appear to be expressed to any appreciable extent in adipose tissue or other lipogenic tissue.

Herein, the ACC-*α* is discussed as it is involved in the *de novo* synthesis of fatty acids. ACC-*α* has been cloned and characterized in depth in many species including human [33], rat [34], cattle [35], chicken [36], yeast [37] and bacteria [38]. In all cases the translated product is a 2,346 aa protein with high similarity among species. Table **1** summarizes the main differences among the characterized species.

In sheep there is no exception from the rule, according to Barber and Travers [39]. The ovine ACC cDNA, encodes a protein of 2,346 aa residues with a calculated molecular mass of 265 KDa. The deduced amino acid residues sequence shows a well conserved protein among mammals and other species. Moreover, heterogeneity in the 3’ UTR was observed, revealing in two transcripts with 2,065 or 1,635 nt. This is due to the differential use of two polyadenylation sites. Concerning the region of 5’ UTR, similar to all determined species, four identified clones have been described, falling into two classes of transcripts; Class 1, which differ in the presence or absence of a 47 nucleotide sequence and Class 2, which differ in the presence or absence of a 61 nucleotide and / or a 47 nucleotide sequence [40]. Class 1 transcripts are found in liver and adipose tissue under lipogenic conditions, while Class 2 transcripts are found in all tissues and are increased in abundance in mammary gland during lactation. The same characteristics are also reported for the bovine counterpart [35].

The ACC-*α* isoforms are products of two promoters, P1 and PII, which are acting in a tissue-specific manner. The use of these promoters results in the generation of the observed heterogeneous population of mRNA containing, the primary coding exon (Fig. **2**). In contrast to other species, Barber and Travers [29] reported the presence of a novel transcript of ACC-*α* gene in the mammary gland. According to the authors, this transcript (termed 5A/ E5A-type transcript) is a product of a third promoter (PIII) resided inside intron 5 [35, 40] and differs from the previously described mRNAs in that exon 5 is replaced by a 424 nt sequence representing the 5’ terminus of the mRNA [40]. Additionally, its expression is tissue-restricted; it is undetectable in adipose tissue, spleen, heart and skeletal muscle, while is present in liver, kidney, lung, brain, and is observed in very high levels in lactating mammary gland.

According to mRNA measurements of ACC-*a* transcripts, in subcutaneous adipose tissue from non lactating sheep approximately the 60% is derived from PI activity, while lactation results in a 88% reduction in total PI transcripts. In the contrary, it reduces the total level of transcripts PII by only 50% [41]. This transcript diversity, according to Travers and Barber [41] is due to insulin-glucocorticoid interactions in ovine adipose tissue. Concerning PIII promoter activity, expression of E5A transcripts are increased 15-fold relative to mammary tissue of non-pregnant non-lactating animals. Generally, E5A mRNAs contribute less than 10% of the total ACC-*α* mRNA in most tissues that is expressed, but only in lactating mammary gland it exceed 30% of the total [29].

Promoter PI and PIII have been well characterized in sheep by previous researchers [29, 40, 42]. PI promoter revealed the presence of one TATA box, two E-box motifs and a CAAT box motif. Analysis of the PI promoter using deletion / mutation constructs, showed that the most close E-box motif to the transcription start site confer response to insulin. In this motif the transcription factors USF-1 and USF-2 were observed to bind, acting as an insulin-response sequence [40].

Concerning the PIII promoter, which is only observed in ruminant species [35, 40], it lacks both TATA and CCAAT boxes proximal to the transcription start site, having instead a sequence homologous to the *inr* sequence CTCANTCT, found in a large number of TATA-less promoters [43]. Additionally, consensus sequences for binding a number of transcription factors including two E-boxes, an inverted CCAAT box element (ICE) and a STAT motif were noted. Deletion analysis conducted in the promoter region showed that E-box and ICE results in a 75-95% reduction of the promoter activity. Moreover, SREBP-1 found to regulate the activity of PIII promoter between non-lactating and lactating mammary gland in a more extensive rate than that of USF-1, -2, and NFY transcription factors, which bind at the identified E-boxes [42]. The STAT motif represents a sequence where the prolactin receptor can bind and confer response to stimuli concerning prolactin signalling. According to Mao *et al.* [44], in the bovine counterpart (PIII promoter) the STAT5 transcription factor can bind to this site leading to an induction of promoter activity by prolactin and dexamethasone. Additionally, the PII promoter is also active in mammary gland and plays a crucial role for milk fat synthesis in sheep [45]. According to Barber *et al.* [28] the synergistic signaling between glucose and insulin increase the activity in PII promoter and is depended on two Sp1 motifs that flank two SRE-half sites, where SREBP transcription facror (1a, 1c) can bind. These findings emerge the importance role of SREBP-1 in the regulation of the *de novo* synthesis of fatty acids in lactating mammary gland. Moioli *et al.* [46] reported three single nucleotide polymorphisms (SNPs) in the PIII promoter of ACC-*α* gene in four Italian sheep breeds; Gentile di Puglia, Sopravissana, Comissana and Sarda. The variant alleles that were identified were G_1330_➔T, C_1338_➔G and C_1430_➔T. Allele frequencies of Sarda breed found to differ significantly with respect to the other considered breeds. Association of such polymorphism with productivity traits would be a further implication.

Apart from the ovine ACC-*α* gene, the goat counterpart has been also characterized. Travers and Barber [47] have reported the isolation of a partial cDNA clone (834 bp) coding the ACC-*α* for goat. The isolated sequence, according to the authors, corresponds to about 11% of cDNA and revealed over a 94% of amino acids identity with respect to rat and chicken counterpart. Recently, Badaoui *et al.* [48] reported the molecular characterization of the goat counterpart. The sequence covers the 78% of the coding sequence and partially encompassed exons 3 to 46. The encoded protein shows over a 99% identity with its ovine and bovine orthologs. At transcriptional level, Travers and Barber [47] tried to assess the effects of different milking frequency on ACC gene expression in lactating goat mammary gland. The prolonged thrice daily milking found that produces a marked increase in the amount of ACC mRNA/mg DNA compared with a twice daily milking (41,0 ± 3,2 vs. 16,6 ± 6,5, P<0.01) indicating that increased ACC enzyme activity associated with the thrice daily milking is in part due to an increase in the abundance of the corresponding mRNA. Badaoui *et al.* [48] managed to identify a segregating silent single nucleotide polymorphism (SNP) at exon 45 of goat ACC-*α* gene (C_5493_➔T) in four Spanish breeds (Murciano-Granadina, Teramana, Majorera and Malaguena). Association of this SNP with milk traits showed that the specific C_5493_➔T mutation is associated with fat yield, lactose content and somatic cell count.

### 3.2. Fatty Acid Synthase (FAS)

Fatty acid synthase (FAS) is a key enzyme in the lipogenic pathway that catalyzes all the reactions involved in the last step of the fatty acid biosynthetic pathway and concerns the conversion of acetyl-CoA and malonyl-CoA to palmitic acid. FAS represents one of the more complex multifunctional polypeptide structures discovered so far to date, because a single polypeptide contains all the catalytic components required to a series of 37 sequential reactions, leading to the formation of palmitic acid. Much of the knowledge concerning FAS gene and its regulation comes from studies conducted on rodents, human and avian species [for review see  49, 50]. The gene has been cloned and characterized in rat [51], human [52], goose [53] and chicken [54].

The enzyme in eukaryotes consists of two identical polypeptides of approximately 2,500 residues, it appears as homodimer and each subunit has a molecular mass of 270 KDa. The amino sequences show high identity among the characterized species which reach the 80%. The active enzyme is organized in a head to tail fashion, generating two active catalytic centers, containing seven catalytic activities and an acyl carrier protein (ACP) [52].

Concerning ruminant species, the only known data about FAS, is the recently cloned bovine counterpart [55]. The gene consists of 43 exons which together with the flanking regions were found to be evolutionary conserved. Moreover, at cDNA level in contrast to other characterized species, two transcripts were identified; one long form which consists of a 7,542 bp coding region which encodes a protein of 2,513 aa residues, and a 358 bp shorter form, which derives from an alternative splicing of exon 9 resulting in a premature termination codon. Additionally, the gene maps to 19q22 chromosome region [56] and it is expressed higher in brain, testis and adipose tissue than in liver and heart. Although the bovine FAS gene is expressed in all tissues, a different expression pattern of the two transcripts has been observed. FAS-1 transcript is only detectable in tissues with the highest fatty acid synthesis in ruminants, leading to the suggestion that in tissues where both transcripts are observed FAS gene may be regulated by the ratio between the two transcripts.

The promoter region of the gene in ruminants consists of one TATA and CCAAT box with several potential binding sites for the nuclear transcription factor SP1 [57], following the same pattern as in rat [51]. However, in rat counterpart binding sites for fatty specific element (FSE), estrogen response elements, thyroid hormone, growth hormone, progesterone and glycocorticoide response were observed. To our knowledge there is no such report for ruminants counterparts as similar studies have not yet conducted. However, Roy *et al.* [57] observed a substitution of G➔C in the untranslated exon 1 of the gene, altering the putative SP1 transcription factor binding site and hence, leading to a less stable folding of the 5΄ UTR. Additionally, an A➔G substitution in exon 34, which leads to a replace of threonine by alanine, is associated with increased milk-fat content in Holstein-Friesian cattles. Travers and Barber [47], using a heterologous (human) probe for FAS gene, managed to determine the effect of the prolonged daily milking in the expression of the goat counterpart mRNA. As they reported, the prolonged thrice daily milking produced a marked increase in the amount of FAS mRNA/mg DNA compared with twice daily milking (114 ± 23 vs. 53,4 ± 14,3, P<0.01) and this paralleled the increase observed for FAS enzyme activity /mg DNA (774 ± 75 vs. 453 ± 85, P<0.01), indicating that the increased ACC enzyme activity associated with the thrice daily milking is in part due to an increase in the abundance of the corresponding mRNA. Although, these studies may offer new insights in the regulation of FAS gene, ovine and goat counterparts should be isolated and cloned in order to elucidate useful information about FAS gene and protein in small ruminants.

### 3.3. Genes Encoding NADPH Generating Dehydrogenases

Many studies have been conducted concerning NADP dependent dehydrogenases and their enzymatic reaction in different stimulus in various species [14, 24, 58-61]. According to Strutz and Rogdakis [58] the levels of NADPH generating dehydrogenases form a valuable criterion to assess the general lipogenic enzymatic activity in swine. Using this criterion, Rogdakis *et al*. [62] made it possible to develop an efficient selection program on backfat content in swine. Moreover, a synchronized enzymatic activity of the NADPH producing enzymes has been reported in ovine adipose tissue [14, 24] suggesting a possible use of these enzymes as biochemical markers of fat deposition and a potential involved mechanism of control at molecular level.

#### 3.3.1. Glucose 6-Phosphate Dehydrogenase (G6PD)

Glucose 6-phosphate dehydrogenase is the rate-limiting enzyme of the pentose phosphate pathway, catalyzing the first committed reaction. It is responsible for the conversion of glucose 6-phosphate to 6-phosphogluconate reducing NADP to NADPH, which is further used in biochemical procedures of the cell such as the *de novo* fatty acid synthesis or the protection of cell against the oxidative damage *via* the diminish of reduced glutathione (GSH). The importance of the enzyme was elucidated when its enzyme activity was associated with deficient cases in human (for review see 62). Moreover, recently, Tien *et al*. [63] showed that suppression of G6PD activity led to diminished proliferation of several cell lines. Previous studies have, also, revealed elevated G6PD activities in malignant tissues in various tissues, suggesting a correlation between G6PD and cancer. However, several epidemiology studies did not reveal any difference between G6PD-deficient and healthy patients, but it is true that deficient individuals may suffer from an increased risk of diabet and cataract.

The enzymatically active form of G6PD is either a dimmer or a tetramer of a single protein subunit of 514 amino acid residues with a molecular mass of 59,096 Da and contains tightly bound NADPH for almost all the species [64]. Three are the critical regions for enzyme function which are also highly conserved in all higher mammals: i) the substrate binding site which is located near the Lys205 ii) the catalytic NADP binding site, which is located near the N-terminus and is important for its catalytic action and iii) the structural NADP binding site, which is important for the stability of the enzyme. At 3D-structural level the protein appears to form a classic dinucleotide binding fold and a domain following a *β+α* fold (Fig. **4**), which is characteristic of all G6PD proteins analyzed so far.

At molecular level, G6PD has been well studied in many eutherian monogastric species. Complete characterized sequences have been already published for human, mouse and rat genes including promoter regions [65-69], while many studies have shown gene’s regulation in respect to various stimuli [for review see 70]. In all species studied so far G6PD is considered as an X-linked gene displaying a “housekeeping” profile [64, 70]. In human the gene spans 18Kb containing 13 exons and maps on Xq28. Moreover, the number and the size of exons/introns and the sequence of exons are conserved in higher eukaryotes. The sequence identity between human G6PD cDNA and that of mice or rats reaches the 90%. The similarity between the mouse and the rat cDNA sequence is even higher (95% identity). It should be noted that the structure of the gene is unusual in that the second intron is too long (11 Kb in hunam) and accounts for almost half the gene. The large size of second intron is also conserved between human, rat and mice. The promoter of G6PD embedded in a CpG island that is conserved in higher eukaryotes. It also contains a TATA-like sequence (TTAAAT) and numerous stimulatory protein 1 elements (SP1 sites), but no CAAT element. A wide number of mutations (over 140) have been found in the coding region of human coding region (single nucleotide mutations, small deletions, spliced mutations). In most cases these mutations cause G6PD deficiency by decreasing the *in vivo* stability of the protein. Although Menousos *et al*. [71] tried to associate promoter G6PD mutations with deficient cases, no correlation was found.

**Fig. (3):**
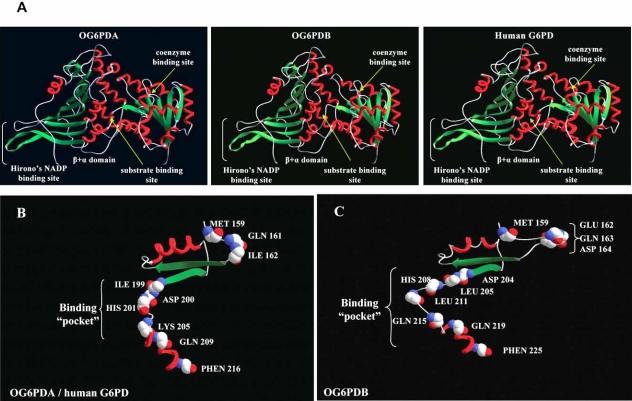
Ovine G6PD protein model [72]. (A) Theoretical three-dimensional molecular model of ovine G6PD protein (left and centre figure) and human protein (right figure). The colored segments of the backbone structure mark the location of α-helix (red), β-sheet (green) and coil (white). (B) Structural representation of catalytic motif of OG6PDA isoform and human G6PD protein. (C) Structural representation of catalytic motif of OG6PDB isoforms.

Although in ruminants G6PD is one of the main suppliers of NADPH for the *de* novo synthesis of fatty acids, the only detailed study at molecular lever that has been reported is for the ovine counterpart [72, 73]. Interestingly, according to the authors, two cDNA transcripts, OG6PDA and OG6PDB, were detected encoding for two polypeptides of 515 and 524 amino acid residues, respectively. The formation of OG6PDB transcript is a result of an alternative spliced genomic region originated at the last 31 bp of the intron 5 of the ovine G6PD gene. Both deduced amino acid sequences revealed a well conserved protein containing all the important residues for its catalytic role. Ovine G6PD, like all others G6PD proteins forms a classic dinucleotide binding fold and a domain following a *β+α* fold, containing all the appropriate functional motifs (Fig. **3**). Additionally, the observed extra nine amino acids encoded by OG6PDB isoform cause a frame shift in the polypeptide chain resulting in changes around the area of the potential substrate binding sites. This frame shift, according to the ovine three-dimensioned model of G6PD, causes structural changes in the catalytic binding pocket of the molecule leading to a bigger binding pocket (Fig. **3**). However, OG6PDB isoform contains all the appropriate residues for binding to substrate and catalysis and, thus, it is hypothesized that OG6PDB isoform could probably result in low glucose 6-phosphate dehydrogenase. The interesting is that although OG6PDA transcript showed to follow a “housekeeping” profile in ovine tissues, OG6PDB did not expressed in tissues of low importance for fatty acid synthesis in ruminants (liver, heart, cerebellum), indicating that in tissues where both transcripts are expressed, G6PD may be regulated by the ratio between the two transcripts, depended on the existence stimulus. Additionally, previous studies of genetic linkage analysis using somatic cell hybridization methods showed that ovine G6PD maps to Xq3.8 [74-76] while it appears as a single copy gene in the sheep genome (Laliotis *et al.*, 2007a). It is clear that the investigation of the physiological role of OG6PDB transcript would be of utmost importance in order to clarify the involvement of ovine G6PD gene in lipogenesis.

**Fig. (4):**
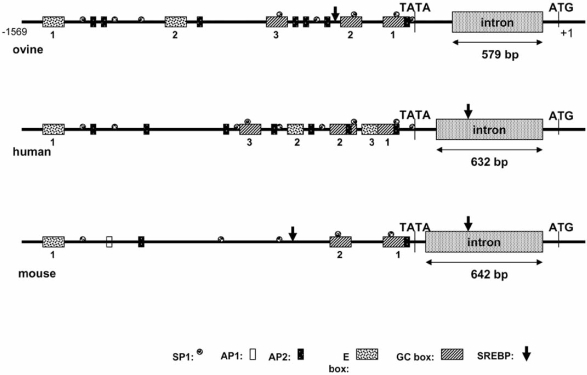
Comparative approach of the 5΄ regulatory region of G6PD gene between ruminant and non ruminant species. The most important motifs such as TATA box, E-box, GC-box, SREBP, SP1 (Stimulating Protein 1), AP1 (Activator Protein 1) and AP2 (Activator Protein 2) are present in the promoter region of the three mammals (sheep, human, mouse). However, some differences were noted. For example, AP1 was only present in murine sequence, E-box 2 and 3 were conserved only in sheep and human regions, while a variation in the location of SREBP binding site was observed among the promoter region of the three analyzed mammals.

At transcription level, ovine G6PD promoter region has many conserved blocks in regard to the other known mammalian G6PD promoter regions [77]. Moreover, the characterization of the region showed the presence of a TATA box, three GC boxes and several binding sites for SP1 and AP2 transcription factors. Many other binding sites for transcription factors involved in lipogenesis (SREBP, USF, RAR, RXR, ROR, HNF4) were also have noted within the 5’ regulatory region of ovine G6PD gene. It is worth mentioning that the most important regulatory elements of ovine G6PD gene (E box, GC box, SREBP, SP1, AP2) are conserved with respect to the human and rodents counterparts (Fig. **4**). However, a slight variation in the number and the position of some of these motifs, such as SREBP, E-boxes and GC boxes, has been reported [77], indicating the first preliminary differences at transcription level of G6PD gene. This observed variation may reflect a different mechanism in the response of ovine G6PD gene, according to various ‘exogenous’ stimuli (i.e. diet rich in carbohydrates, insulin etc.). However, further studies are needed in order to elucidate in depth the roles of these motifs and to better understand the ruminant G6PD regulation and how it influences lipogenesis.

#### 3.3.2. 6-Phosphogluconate Dehydrogenase (6PGD)

The enzyme 6-phosphogluconate dehydrogenase is involved in the third step of the pentose phosphate pathway. It catalyzes the oxidative decarboxylation of 6-phosphogluconate to ribulose 5-phosphate, with the simultaneously release of CO_2 _and the reduction of NADP to NADPH [78].

Eucaryotic 6PGDs are proteins of about 470 amino acids whose sequences are highly conserved. The enzyme is dimeric and NADP dependent for almost all the species [79]. The subunit molecular weight found to be 52 KDa and only the crystal structure of ovine liver enzyme has been determined [80]. The functional importance of the enzyme is generally recognized in providing NADPH for fat synthesis and ribose for nucleic acid synthesis [81]. However, in the last 25 years several studies elucidate the involvement of the enzyme in various human diseases. Thus, 6PGD deficiency has been referred, where the activity of the enzyme was reduced 35% in the affected members of a family under investigation [82]. The 6PGD activity was found to be decreased in aged human erythrocyte populations, with the aged enzyme having 11 fewer lysine residues than the young enzyme. Oxidation of 6PGD may be also considered an important process that takes place during aging of erythrocytes [83].

The amino acid sequences of almost 40 different 6PGDs have been reported including human [84], mouse [85], rat [86] and pig [87] encoding a protein of 482 aa residues. The human 6GPD gene consists of 13 exons, while the size of the introns varies (Table **2**). Introns near the 5’ and 3’ UTR are relative small compared with that existing through out the gene. Interestingly, the number, the position and the size of introns are highly conserved among human, mouse and rat. The amino acid sequence of 6GPD sequence is highly conserved, over 85%, between the higher eucaryotes. Concerning the chromosome map of the gene, it differs between the species analyzed so far. Specifically, human 6PGD maps to 1p36.22, swine 6PGD to 6q22, while murine and rat counterparts map to 4^th^ chromosome and 5q36, respectively.

In ruminants extensive studies have been reported only for sheep. Somers *et al.* [88] determined the amino acid sequence based upon the isolation of the cDNA clones encoding the 6PGD gene in sheep, obtained by a PCR-based strategy. Thus, the isolated cDNA encodes a protein sequence that is 482 amino acid residues long with a molecular mass of 52 KDa. The conservation of the protein sequence is very high as it shares an over 50% similarity with the protein encoded by the *E. coli* 6PGD gene and over 80% similarity with that of other mammals (human, rodents, pig). Additionally, the ovine gene maps to the 12q2.2 chromosome region [74]. Preliminary studies of the genomic structure of ovine 6PGD gene showed that at the 5’ genomic region, the size of introns and exons differed from that reported for human, mouse and rat counterparts, revealing different intron positions, in contrast with the 3’ genomic region, where the size and position was identical to these reported mammalian counterparts. Differentiation of introns / exons size and position is possible related to differentiation in the role of 6PGDH in mammals examined and diversification of the mechanisms related to 6PGDH gene expression [89].

Analyzing in more depth the crystallographic structure studies [80, 90], is revealed the presence of a considerable amount of α-helix (34 % vs. 4% β-sheet). The molecule consists of two domains separated by a “cleft” in which only the smaller domain contains any β-structure. Binding studies with co-enzyme, coenzyme analogues and substrate [91] showed NADP to bind in the “cleft”, distant from the dimmer interface and to have a slightly less open conformation than that of NAD dehydrogenases (i.e. the lactate dehydrogenase ternary complex) [92]. This is dissimilar to NADP-binding sites observed in all other NADP dehydrogenases so far investigated [88].

Concerning the 5’ regulatory region, no sequential information is known so far to date, indicating the need of further research. However, preliminary information can be obtained about transcription of 6PGD gene through studies concerning the expression profile of the 6PGD gene during various productive stages of animals. 6PGD expression profile has been studied in adipose tissue during lactation in two groups of ewes of Chios breed [89]; one with high yield (>1,7 Kg milk / day) and one with low yield (<1,1 Kg milk/ day). It was observed that the expression of 6PGD was higher in the low yield ewes with respect to that of high yield, where the expression was lower. Moreover, as lactation yield became higher, the expression profile of 6PGD gene became lower. This shows that the rate of lipogenesis in adipose tissue is diminished during lactation and with respect to the increase of milk yield.

#### 3.3.3. Cytosolic NADP Malate Dehydrogenase/Malic Enzyme 1 (ME1)

Malic enzyme (or malate dehydrogenase) catalyses the oxidative decarboxylation of malate to pyruvate using either NAD^+^ or NADP as a cofactor. In mammalian tissues three distinct isoforms have been described; a mitochondrial NAD-isoform, and two NADP-dependent isoforms, a first localized in cytosol (ME1) and a second occurred in mitochondria (ME2). Cytosolic malic enzyme (ME1) or NADP-malate dehydrogenase is considered to be a NADPH-donnor for fatty acid synthesis. Also it is involved in the supply of fatty acids with the essential acetyl-coA. As it has been aforementioned, glucose is catabolized to pyruvate and then to acetyl-coA. The last is produced in mitochondria but it is essential for fatty acids biosynthesis which takes place in cytosol [93]. Malic enzyme takes part in the reactions of pyruvate/citrate cycling and thus plays a role in the export of the acetyl-coA to the cytosol. In ruminants, contrary to humans and rodents, the pathway of glucose-pyruvate-acetyl-coA is of little significance, as the principal carbon source for lipogenesis instead of glucose, is the acetic acid produced by the rumen’s microorganisms [94]. The expression of the enzyme is regulated by both hormones and nutrition. In particular, T3 and insulin stimulate malic enzyme in liver and adipose tissue while glucagon blocks this effect. Moreover, a high-carbohydrate low fat diet after a period of starvation results in an increased enzyme’s expression in liver and adipose tissue.

The gene encoding ME1 has been well studied in many monogastric species, including human [95], mouse [96], rat [97] swine [98], duck [99] and pigeon [100] counterparts. The enzyme is a tetramer of four structurally identical subunits each of ~60 kDa. However, the complex appears to be bifunctional as only two of the active sites undergo turnover during catalysis [97].

In sheep, unlike to other species, two transcripts encoding ovine ME1 has been reported [101], which may further elucidate possible explanations for the minor role of cytosolic malic enzyme in these species. The transcripts share the same CDS, but they differ in the length of 3’ UTR (Table **3**), which is a result of a dual distinct polyadenylation signals. Such types of transcripts have been also reported for ME1 murine [96] and swine [98] counterparts. Concerning the 5’ UTR, is common for the two transcripts and is also highly GC% rich, like in human and rodents. All deduced amino acids sequences analyzed so far today are highly conserved, showing an over 75% identity (Table **3**), and revealing the important biological function of the enzyme in living organisms.

Concerning the protein structure, two studies, based on crystal forms from pigeon [102] and rat [103] cytosolic NADP^+^ malate dehydrogenase, have been reported. According to them, four major conserved regions are distinguished: a) the divalent cation binding residues b) the substrate binding residues, c) the NADP^+^ cofactor binding residues and d) the catalytic residues. Crystal structure comparison of human NAD(P)^+^-dependent malic enzyme and the pigeon cytosolic malic enzyme shoed that the backbone traces are similar with small local conformational differences, reflecting the structural basis of the different properties observed in the catalysis of substrate or cofactors specificities [104]. Alignment of the cytosolic malic enzyme proteins of sheep and pigeon revealed that the overall structure of the proteins is similar [101].

At transcription level, available information concerns the human, rat and ovine promoter region. The molecular cloning and functional characterization of the human ME1 promoter region showed that the region is GC rich, it has multiple transcription start sites and lacks of TATA or CCAAT boxes [105]. Similar characteristics have been reported for the rat counterpart [106]. The presence of an inverted T3 response element (TRE), where thyroid receptor beta (TRbeta) can bind in the absence of T3 in human ME1 promoter, represses its promoter activity, revealing that ME1 transcription is very responsive to thyroid hormones.

Concerning the ovine ME1 promoter, the same major characteristics as in other species are reported. It is located within a GC-rich region and lacks a TATA-box. According to deletion analysis, a region (−231/−170) that suppressed promoter activity in luciferase assays in HepG2 hepatoma cells but not in 3T3-L1 adipocytes was identified. This region contains a putative triiodothyronine response element (T3RE) that differs from the human ME1 T3RE by two nucleotides. When the human ME1 T3RE was introduced into the ovine ME1 promoter context, transcriptional activity was increased in the hepatic cell lines HepG2 and H4IIE but not in differentiated 3T3-L1 cells. These results suggest that the sequence of the T3RE in the ME1 promoter determines differences in the tissue/species activity of malic enzyme in ruminants and human [107]. This functional difference may be related to the differential contribution of liver and adipose tissue in whole body lipogenesis between humans and sheep as a ruminant species. However, further studies should be conducted in order to support this hypothesis.

#### 3.3.4. Cytosolic NADP Isocitrate Dehydrogenase (IDH1)

Three distinct forms of the enzyme have been found in mammalian organisms including the NAD^+^ dependent IDH, which is located exclusively in the mitochondria, and two NADP-dependent IDH which are found in mitochondria (IDH2) and in cytosol (IDH1), respectively [108-110]. The mitochonrial NAD^+^-dependent isocitrate dehydrogenase catalyzes the oxidative decarboxylation of isocitrate in the tricarboxylic acid cycle, while IDH1 and IDH2 are responsible for the generation of a-ketoglutarate, CO_2_, and NADPH from isocitrate in the cytosol and mitochondria, respectively. NADPH produced in the cytosol is the primary source, as it has been noted, of reducing equivalents utilized for fatty acid synthesis both in ruminants and non ruminant species.

IDH isoforms are encoded by different nuclear genes [111,112] and sequence analysis revealed high levels of homology and amino acid sequence conservation throughout the respective mammalian counterparts [113]. To date, mammalian cDNAs coding for IDH1 (cytosolic) have been isolated from rat [109], mouse [114] and human [113]. Concerning the subgroup of ruminants, the complete characterized sequence has been elucidated for the cattle counterpart [115], while in small ruminants only the ovine cDNA has been cloned and characterized [116].

Ovine IDH1 cDNA is 2,254 bp long and it consists of 166 nt 5’ UTR, 1,245 nt coding region, 843 nt 3’ UTR and poly A tail [116]. It is worth of mentioning that no profound polyadenylation signals has been found on the ovine IDH1 sequence, which coincides with the observation of Nekrutenko *et al.* [113] for all the isolated mammalian counterparts. Concerning the deduced protein sequence of ovine IDH1, it consists of 414 amino acid residues, like all other known mammalian counterparts, and has a predicted molecular mass of 46.8 KDa. Moreover, comparison with the all known IDH1 amino acid sequences revealed a well conserved protein throughout evolution. To our knowledge, up to date no extensively study concerning the protein structure of IDH1 has been reported. However, IDH1 gene is present in the ovine genome as a single copy gene, while it maps to the 2q3.4 chromosome region [76], showing a chromosome conservation between the mammalian species.

Kenoutis *et al.* [116] examined the expression pattern of IDH1 in the adipose tissue of growing Chios breed lambs of both sexes, fed with a diet rich in carbohydrate and slaughtered in three different body weights (25 kg, 30 kg and 35 kg). According to the authors, differences in the IDH1 gene expression between male and female animals of the same body weight, animals of different body weight and between fat depositions in the body of the same animal were observed. In males, the expression levels of IDH1 gene in perirenal adipose tissue were higher than those observed in tail and lateral adipose tissue at the weight of 25 kg. As the bodyweight increased, expression became more distinctive. Moreover, lateral adipose tissue demonstrated the highest IDH1 mRNA abundance among the three examined tissues. In contrast, in female lambs, at the body weight of 25 kg, the highest abundance was detected in tail adipose tissue, whereas, as the body weight was increasing, in perirenal adipose tissue.

At transcription level, to our knowledge so far to date no sequential information is available for the 5’ regulatory region of IDH1 gene is ruminants. However, extensive studies have been conducted in the human counterpart. Human IDH1 gene transcription is sterol regulated and its promoter region contains a SRE-sequence element, which is a potential binding site for the SREBP transcription factors. Analysis based on promoter reporter gene, showed that IDH1 promoter is fully activated by SREBP1 and to a lesser extent by SREBP2, while the presence of a CAAT and a GC- box can result in a 6.5% residual activity (Shechter *et al.*, 2003). Based on these data, further studies can been conducted on the identification and characterization of the promoter region of ovine, goat or bovine IDH1 gene, in order to clarify the transcription regulation of the gene in ruminants.

## 4. TRANSCRIPTION REGULATION OF THE LIPOGENIC GENES

The first step of gene expression and the primary step at which gene expression is controlled is transcription. This is succeeded through the recruitment of several transcription factors, which have the ability to bind on certain target-sequences, primarily located in the 5’ upstream regulatory region of the genes, and promote or suppress gene transcription according to the stimulus (i.e. nutrients, hormones etc.). Much of the data concerning the control of lipogenic genes at transcriptional level comes from monogastric animals and especially rodents. To our knowledge this field has not yet be clarified in ruminants. However, in this section we would try to briefly review major data from non ruminant species in order to create a more spherical view about the transcription regulation of lipogenesis.

SREBPs comprise one of the most important transcription factors that can mediate the expression of lipogenic genes. They belong to a large class of transcription factors containing basic helix loop helix (bHLH)-Zip domains. Unlike other members of this class, SREBPs are synthesized as membrane bound precursors that require cleavage by a two proteolytic process in order to release their amino-terminal bHLH-Zip domain into the nucleus to bind to a specific DNA sequence and to activate their target genes in a sterol regulated manner. Moreover, they can be divided into three types: SREBP-2, SREBP-1a and SREBP-1c. The last two are product of a single gene through the use of alternative promoters, in contrast with the former that is encoded by a separated gene. Moreover, SREBP-2 is associated with genes involved in cholesterol metabolism (i.e. LDL-receptor, farnesyl diphosphate synthase, squalene synthase etc.), while SREBP-1 is involved in the regulation of the lipogenic genes such as ACC, FAS, G6PD etc. [for review see 117, 118]. In addition, SREBP-1c regulates direct the lipid homeostasis by activating lipogenic genes including FAS and ACCa genes, which are involved in the production of palmitate. Binding of SREBPs to DNA leads to the recruitment of co-activators such as CBP. However, SREBP-1c has been reported not to strongly interact with co-factors. SREBPs can bind DNA as dimmers at sterol regulatory elements (SREs), which are typically located very near binding sites for SP1 and/or NFY. Moreover, in the majority of promoters, these other elements play an important role in SREBPs function. Thus, SREBPs functionally interact with these additional transcription factors to elevate transcription of the cis-linked genes [118]. The induction of lipogenic genes by insulin and glucose is mediated *via* the action of SREBP-1. This is clearly displayed through the protocol of fasting/refeeding. SREBP-1 can down regulate gene expression when a deprivation of food is applied and the level of insulin is low, while on the other hand it can enhance gene expression when rodents are placed on a high carbohydrate diet. In contrast with glucose and insulin, when a diet rich in polyunsaturated fatty acids is fed, a down regulation of lipogenic gene expression is observed *via* the recruitment of SREBP-1 [119-124].

The presence of E-box motifs in the promoter region of lipogenic genes is of particular importance. This motif (CANNTG) can confer carbohydrate, glucose or insulin response sensitivity [125-127] especially through the binding of SREBP and USF transcription factors on this motif. According to Arkwright-Keeler and Stapleton [128], mutations caused in the unique E-box existing in rat G6PD promoter resulted in significantly reduce of glucose and insulin response. Additionally, the same authors demonstrated that an USF transcription factor which binds to this motif, is involved in the response of G6PD to a high carbohydrate diet. Moreover, mutations that weaken binding of USF-1 and USF-2 to E-box motif, abolish the insulin dependent activation of the fatty acid synthase promoter [122]. Casado *et al.* [129] using mice lacking USF-1 or/and USF-2 showed the importance of these transcription factors in mediating the stimulatory effect of insulin/glucose on fatty acid synthase expression. In addition to the E-box motif, the carbohydrate response transcription factor (ChoRF) can also confer response to glucose stimulus. Its binding site is appeared as a combination of two E-box represented by the palidromic sequence CANNTGn_5_CANNTG and it has been observed in the promoter regions of many lipogenic genes such as fatty acid synthase, pyruvate kinase or S14 gene. According to Rufo *et al.* [130], the ChoRF appears to work in conjunction with SREBP-1c and USF to impart glucose and insulin responsiveness to the fatty acid synthase promoter.

Additionally, much discussion has been made about the PPARγ transcription factor and its impact on the regulation of lipogenic genes. It should be noted that this transcription factor is part of the adipocyte differentiation program concerning the field of adipogenesis [for review see 131, 132] and it has little to be associated with the regulation of lipogenesis. However, according to Kersten [122] patients who took synthetic PPARγ activators frequently gain weight, something which implies that PPARγ apart from adipogenic effect can also have a lipogenic effect. In addition, PPARs, are considered as monitors of the oxidized lipids, as fatty acids are feed-forward regulators of PPARs. However, PPAR activation leads to the induction of many genes involved in fatty acid oxidation or fatty acid storage [118]. Recently, much ground on the transcription regulation of lipogenic genes have gained some members of the nuclear receptor family such as RAR, RXR, ROR and HNF4. Nuclear receptors function as ligand-activated transcription factors that regulate the expression of target genes to affect various processes. These transcription factors have been reported to modulate and control the transcription of genes involved in lipogenesis, causing activation or repression of transcription [133-136]. They can bind on the recognition site either as homodimers or heterodimers, i.e. RAR/RXR influencing the transcriptional control of the lipid-sensing pathways, in which they are involved [134]. In addition they can act in a more indirect way, favoring or not the presence of other regulatory proteins which control the transcription regulation of lipogenesis. For instance, RXRa and HNF4 favor the presence of SREBP-1c resulting in up- or down-regulation of lipogenic genes [136].

It is clear that although many mechanisms controlling the transcription regulation of lipogenesis has been fully or partially elucidated, much data, especially concerning ruminants, has to be clarified. As ruminants demonstrate certain differences with regard to fatty acid synthesis and lipogenesis, implicating a potential different way of controlling the genes involved in this procedure, further investigation on transcription regulation of lipogenic genes in ruminants, may offer new insights in this type of control.

## WHAT CAN BE DONE IN THE FUTURE?

Undoubtedly, years of intensive study in mammalian species have definitely identified several key pathways and molecules that are involved in the regulation of the *de novo* synthesis of fatty acids. Many advances have also been conducted in ruminants revealing the differentiation of some aspects in comparison to other mammalian species with respect to lipogenesis. The application of molecular biology elucidated many unexpected complexities, such as multiple promoters, different isoforms of enzymes (G6PD, FAS, acetyl-CoA carboxylase, malic enzyme) and many single nucleotides mutations. Despite these advances, there are still many questions that remain unsolved, ranging from the association of single nucleotide mutations with productivity traits to the very challenging aspect of manipulating lipogenesis.

Investigation of the observed mutations at DNA level and their association with productivity traits i.e. milk fat content, carcass quality/marbling, would be a first approach in manipulating lipogenesis in farm animals. This association can further be used in selective breeding schemes so that new lines with the desired characteristics, such as less fat content, could be achieved. In addition, the discover of novel mutations on DNA level may also reveal new mechanisms of transcript regulation. Moreover, the elucidation of the physiological role of new identified transcripts of genes i.e. ovine G6PDB transcript or ovine ME1 transcripts that are involved in lipogenesis in ruminants and are not found in other species, is of particular importance as they may offer novel data in the mechanisms of regulation of the *de novo* synthesis of fatty acids according to various stimuli in comparison to human and rodents.

Additionally, identification of common regulators of lipogenic genes could offer new prospects. A working model of this aspect could be the “platform” of genes encoding NADPH generating dehydrogenases. Specifically, previous studies [14, 24] report a synchronized enzymatic activity of the NADPH producing enzymes in ovine adipose tissue. This observed synergic action of the mentioned dehydrogenases may be due to involved mechanisms at transcriptional level, using a common regulator. The identification of such a mechanism may probably permit the control of lipogenesis in specific tissues. For example, interfering in such a mechanism using i.e. an inhibitor, can lead to the reduction of mRNA coding for the respective lipogenic enzymes and hence could lead to a tissue specific reduction in the amount of lipogenic proteins and a diminution in fatty acid synthetic capacity.

Transgenesis may be offer many advances in the field of lipogenesis as it is considered a potential experimental approach for investigating both the control of the *de* *novo* synthesis of fatty acids and for modulating the improvement of animal performance. Such case studies have been limited in rodents. For example, in mice lacking lipoprotein lipase fatty acid composition of adipose tissue lipids indicated a compensatory increase in *de novo* fatty acid synthesis [137]. To our knowledge, transgenic animals concerning the main lipogenic genes have not been reported. However, Ha and Kim [133] reported the production of an adipocyte cell-line in which the amount of ACC was reduced using a ribozyme construct resulting in a decreased rate of fatty acid synthesis. Despite the fact that in ruminants no transgenic effort has been conducted, the development of proper adipocyte cell lines, where *in vitro* studies can be conducted, would be an advantage compensating the large generation interval observed in ruminants in contrast with rodents.

## Figures and Tables

**Table 1:** Main Characteristics of ACC-*α* Gene in Various Species

Species	Chromosome Localization	Coding Exons	Amino Acids	Coding Sequence
*Homo sapiens*	17q21	54	2346	245 Kb
*Mus musculus*	11	54	2345	206 kb
*Rattus norvegicus*	10q26	54	2345	195 Kb
*Ovis aries*	11	??*	2346	7401 bp
*Sus Scrofa (partial)*	12p13	??*	??*	??*
*Bus taurus*	19q13	??*	2346	??*
*Gallus gallus*	19	52	2324	>91.5 kb

* It has not been yet determined.

**Table 2:** Intron and Exon Barriers in Human 6GPD Gene

EXON		Coding EXON		INTRON	
*coords*	*length*	*coords*	*length*	*coords*	*length*
1 - 98	98 bp	91 - 98	8 bp	99 - 601	503 bp
602 - 677	76 bp	602 - 677	76 bp	678 - 1365	688 bp
1366 - 1545	180 bp	1366 - 1545	180 bp	1546 - 4043	2498 bp
4044 - 4109	66 bp	4044 - 4109	66 bp	4110 - 5133	1024 bp
5134 - 5252	119 bp	5134 - 5252	119 bp	5253 - 9043	3791 bp
9044 - 9113	70 bp	9044 - 9113	70 bp	9114 - 12390	3277 bp
12391 - 12525	135 bp	12391 - 12525	135 bp	12526 - 14034	1509 bp
14035 - 14224	190 bp	14035 - 14224	190 bp	14225 - 17959	3735 bp
17960 - 18090	131 bp	17960 - 18090	131 bp	18091 - 18348	258 bp
18349 - 18482	134 bp	18349 - 18482	134 bp	18483 - 19798	1316 bp
19799 - 19898	100 bp	19799 - 19898	100 bp	19899 - 20389	491 bp
20390 - 20512	123 bp	20390 - 20512	123 bp	20513 - 20602	90 bp
20603 - 21117	515 bp	20603 - 20722	120 bp		

**Table 3:** Structure of Ovine Malic Enzyme 1 and Differences between Species

ME 1	Full length	Coding sequence	5’ UTR	3’ UTR	Similarity			
					species	CDS	protein	
*Transcript 1*	2121 bp	1716 bp / 571 a.a.	58 bp	346 bp	human	90%		96%
					swine	91%		97%
*Transcript 2*	3243 bp			1469 bp	rodents	84%		93%
					chicken	77%		78%

## ABBREVIATIONS

aa: = Amino acids
ACC: = Acetyl-CoA carboxylase
bp: = Base pair
cDNA: = Complementary to RNA
CDS: = Coding sequence
G6PD: = Glucose 6-phosphate dehydrogenase
FAS: = Fatty acid synthase
IDH: = Isocitrate dehydrogenase
6PGD: = 6-phosphogluconate dehydrogenase
kDa: = Kilodalton
ME: = Malic enzyme
NADP: = Nicotinamide adenine dinucleotide phosphate
NADPH: = Reduced nicotinamide adenine dinucleotide phosphate
nt: = Nucleotide
PCR: = Polymerase chain reaction
